# Catalysts for CO_2_/epoxide ring-opening copolymerization

**DOI:** 10.1098/rsta.2015.0085

**Published:** 2016-02-28

**Authors:** G. Trott, P. K. Saini, C. K. Williams

**Affiliations:** Department of Chemistry, Imperial College London, Exhibition Road, London SW7 2AZ, UK

**Keywords:** catalysis, polycarbonate, ring-opening copolymerization, CO_2_

## Abstract

This article summarizes and reviews recent progress in the development of catalysts for the ring-opening copolymerization of carbon dioxide and epoxides. The copolymerization is an interesting method to add value to carbon dioxide, including from waste sources, and to reduce pollution associated with commodity polymer manufacture. The selection of the catalyst is of critical importance to control the composition, properties and applications of the resultant polymers. This review highlights and exemplifies some key recent findings and hypotheses, in particular using examples drawn from our own research.

## 1. Introduction

**Figure 1.**
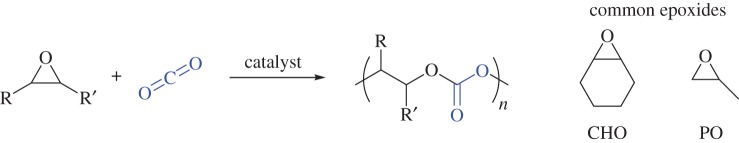
The ROCOP of carbon dioxide and epoxides to produce aliphatic polycarbonates. (Online version in colour.)

The ring-opening copolymerization (ROCOP) of carbon dioxide and epoxides is an interesting method to synthesize a range of aliphatic polycarbonates (figure 1) [1–5]. The reaction was discovered more than 40 years ago and has since continued to attract attention as a means to reduce pollution associated with polymer manufacture and to ‘add value’ to carbon dioxide [1]. It should be made clear that the use of CO_2_ in any chemical manufacturing process is unable to make a large impact on overall CO_2_ levels in the atmosphere. However, it remains important to pursue CO_2_ utilization as a means to reduce emissions, particularly those associated with existing, large-scale industrial processes and as an economic driver to support carbon capture [6,7]. The ROCOP process is strongly dependent on the selection of the catalyst, with various homogeneous and heterogeneous catalysts having been reported [5,8–15]. The focus for this review article will be to highlight and exemplify some of the key findings in this area of catalysis, in particular using examples drawn from our own research. The intention is not to provide a comprehensive review of all known catalysts; indeed, such reviews are already available [5,8–15].

The ROCOP reaction is a rare example of a truly catalytic process with the potential to deliver large-scale quantities of product, which genuinely consumes carbon dioxide. The most commonly studied epoxides are cyclohexene oxide (CHO) and propylene oxide (PO). Depending on the epoxide and the selectivity of the catalyst, up to 31% (polycyclohexene carbonate) or 43% (polypropylene carbonate) of the polymer mass derives from CO_2_. The primary application for the polymer products is as low-molecular-weight (*M*_n_), (poly)hydroxyl-terminated ‘polyols’, which are widely used in the manufacture of polyurethanes [16]. Polyurethanes themselves are applied as flexible/rigid foams, adhesives, coatings and elastomers, as well as in many other areas. It has been shown that the properties of CO_2_-derived polyols are suitable to replace polyether polyols in some applications [16,17]. Given that polyether polyols are prepared by epoxide homopolymerization, the ROCOP of CO_2_ and epoxides can also be viewed as a means to ‘replace’ a substantial portion of petrochemically derived resource (epoxide) with a renewable one (CO_2_). A recent detailed life-cycle analysis study compared these two types of polyols, showing significant reductions (approx. 20%) in both fossil resource depletion and greenhouse gas emissions for the CO_2_-derived polymers [18].

Furthermore, the replacement of fossil-derived epoxides is economically attractive and is stimulating a number of commercialization studies [16,19] (http://www.empowermaterials.com/; http://www.novomer.com/; http://www.covestro.com/en/Sustainability/Productions/Polyols; http://www.econic-technologies.com/ [accessed 4 September 2015]). It has also recently been demonstrated that ROCOP catalysts are compatible with carbon capture and storage (CCS) processes [20]. Studies have shown that some homogeneous magnesium catalysts can be used in polymerizations where the carbon dioxide is captured at a CCS demonstrator plant attached to a UK power station. The catalysts showed near-equivalent performances using such captured gases compared to using ‘pure’ carbon dioxide. Furthermore, the catalyst showed a high tolerance to various impurities present in captured CO_2_, including water, N_2_, CO, thiols and amines [20].

## 2. Polymerization pathways and mechanisms

**Figure 2.**
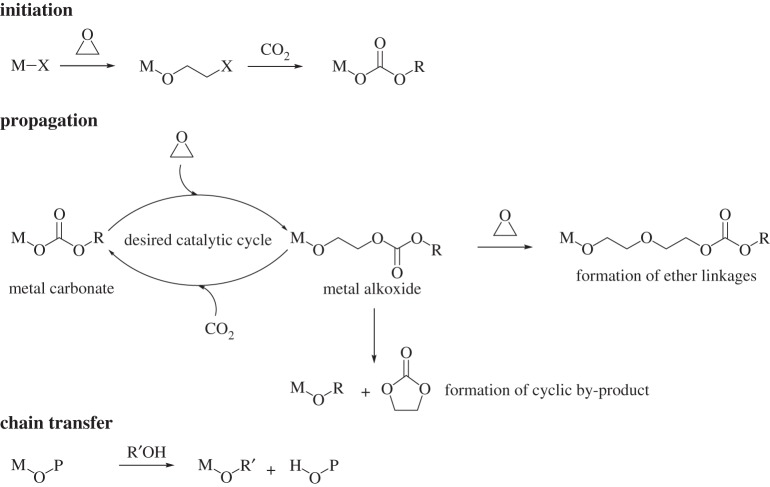
Catalytic cycle of CO_2_/epoxide copolymerization.

A range of different catalysts are known but all catalysts contain metals, with Zn(II), Co(III) and Cr(III) being particularly common [5,8–14]. Prior to an examination of these catalysts, it is worth considering the series of reactions that are proposed to occur at the metal active site during polymerization, the major ones of which are illustrated in figure 2 [3,11]. The polymerization is initiated by coordination of an epoxide molecule and the subsequent ring opening by the nucleophilic attack of a carbonate group or ligand (X), so as to form a metal alkoxide intermediate. During chain propagation, carbon dioxide inserts into the metal alkoxide intermediate to form a metal carbonate species. The metal coordinates another molecule of epoxide, and nucleophilic attack by the carbonate group leads to the ring opening of the epoxide and formation of a new metal alkoxide species. Propagation therefore involves the ‘cycling’ between metal alkoxide and carbonate intermediates. The polymerization is terminated by exposure to conditions/reagents that lead to hydrolysis of the growing polymer chain and formation of a polymer chain end-capped with a hydroxyl group.

There are also side reactions within this process, the proportions of which depend on the conditions, substrate and catalyst selected. The formation of ether linkages in the polymer chain can occur due to the occurrence of a metal alkoxide attack on an epoxide molecule instead of CO_2_ insertion. Such linkages change the polymer properties, which may be beneficial depending on the application, but nevertheless reduce the CO_2_ sequestered in the polymer backbone [16,17].

Five-membered ring cyclic carbonate by-products can also form; indeed, this is the thermodynamic product of the reaction between CO_2_ and epoxides and thus favoured under forcing conditions. The cyclic carbonates can form by depolymerization or ‘back-biting’ reactions; the extent of these depend on the catalyst and the ceiling temperature for the given polymer [21,22]. The cyclic carbonates can also form ‘off-metal’ and are particularly common, and sometimes problematic, contaminants when co-catalysts or ionic additives are applied [5,13]. For some types of catalyst, most commonly with metal salen catalysts, it is proposed that the addition of ionic co-catalysts leads to the polymer chains being in equilibrium between metal coordination and ‘free’ anionic polymer chains [5]. Such ‘off-metal’ chains have been shown to undergo cyclization to form cyclic carbonate by-products [23].

Chain transfer reactions also need to be considered. These reactions occur when the polymerizations are conducted under so-called ‘immortal’ conditions in the presence of protic compounds, such as alcohols, amines and water, among others [24–27]. When such protic reagents are present, the metal-alkoxide-terminated polymer chain is proposed to be in rapid exchange with the protic reagent, generating metal alkoxides and ‘free’ hydroxyl-terminated polymer chains. The chain transfer processes are proposed to occur more rapidly than propagation, leading to highly controlled polymerizations where the *M*_n_ of the polymer is determined by both the catalyst and chain transfer agent (protic reagent) concentrations. It is worth noting that in ROCOP the polycarbonate *M*_n_ is commonly experimentally observed to be rather lower than those expected for living polymerizations (where the *M*_n_ would depend only on the catalyst concentration) [25]. This is due to the presence or formation of chain transfer agents, such as diols, in the epoxide monomers used [28–32]. In fact, the exploitation of catalysts able to operate under immortal polymerization conditions, i.e. where such chain transfer agents are added in larger excess compared to the catalyst, is essential to selectively prepare the polyols that are required in polyurethane manufacture [18,20,24].

The catalysts for ROCOP have some general features: the metals are Lewis acids; the metals’ redox reactivity should be limited; the metal alkoxide and carbonate intermediates are labile; and initiating ligands (X) include alkoxides, carboxylates, halides and other anionic groups [5,8–14]. It is common in this area of catalysis that dinuclear or bimetallic catalysts show good performances and bimetallic pathways are proposed to accelerate epoxide ring opening [10]. Finally, the polymerization catalysts should ideally be colourless, odourless, inexpensive and have low toxicity, as they may contaminate the polymer product. In this context, there have been a number of reports of strategies to remove and recycle homogeneous catalysts [33–35].

## 3. Heterogeneous catalysts

The two major classes of heterogeneous catalysts are zinc glutarate (or other carboxylates) and double metal cyanides [4,36–52]. Both are well known, and in some cases industrially applied, as epoxide homopolymerization catalysts [52]. For copolymerizations using carbon dioxide, these heterogeneous catalysts require much more forcing conditions than homogeneous species. In particular, high pressures of carbon dioxide are necessary, and perfectly alternating enchainment does not occur, but rather ether-enriched polycarbonates are produced.

**Figure 3.**
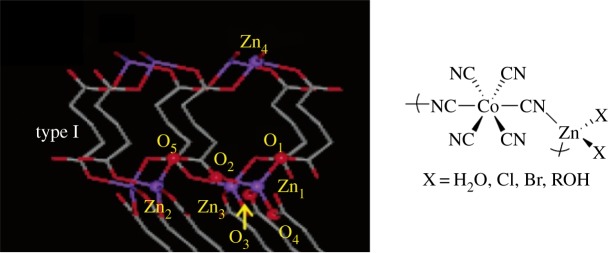
One of the crystal structures for zinc glutarate and a generic illustration of the structure of the repeat unit in zinc-cobalt double metal cyanide catalysts. Adapted with permission from [38]. Copyright (2004) American Chemical Society. (Online version in colour.)

Zinc glutarate has been extensively studied, and two features stand out that increase activity: (i) the addition of ethylsulfinate groups and (ii) an increase in crystallinity of the catalyst [4,36–44]. The most commonly studied double metal cyanide catalyst is Zn_3_(CoCN_6_)_2_ (figure 3), although mixed Zn/Fe(III) species are also reported. These catalysts are typically applied as part of mixtures with so-called ‘complexation’ agents, including salts, alcohols and solvents [45–52]. They generally show very high activities but low CO_2_ uptakes; indeed, they commonly yield poly(ether carbonates) rather than perfectly alternating copolymers. Studying the mechanisms of such heterogeneous catalysts is very challenging; nevertheless, theoretical and experimental studies have led to the proposal that bimetallic polymerization processes are important. In particular, one study proposes that the active sites in zinc carboxylates should be separated by 4–5 Å [44].

## 4. Homogeneous catalysts

The common homogeneous catalysts can be classified into two broad types:

(a) *Bicomponent catalyst systems* comprising metal(III) complexes used with co-catalysts. The catalysts are usually complexes of Co(III), Cr(III), Mn(III) or Al(III) coordinated by ligands such as salens or porphyrins. The co-catalysts are typically ionic compounds, the most common of which is bis(triphenylphosphine)iminium (PPN) chloride (PPNCl), or Lewis bases, commonly 4-dimethylaminopyridine (DMAP).
(b) *Dinuclear or bimetallic catalysts* comprising metal(II/III) complexes. Most commonly these are complexes where two metals are coordinated by tethered ‘mononucleating’ ligands, such as Zn(II) β-diiminates (BDIs) or tethered Co(II)/Cr(III) salens. There are also examples of deliberately dinucleating ligands, such as macrocyclic ligands coordinated to Zn(II), Mg(II), Co(II/III) or Fe(III).

Most homogeneous catalysts operate under moderate/high pressures of CO_2_, and usually require more than 10 bar pressure, but, in contrast to heterogeneous catalysts, they do yield highly alternating copolymers [15,53–57]. In a drive to change the polymerization process conditions, catalysts have now been developed that show high activities and perfectly alternating enchainment under low pressures of carbon dioxide, including at 1 bar pressure [58–60].

### (a) Bicomponent catalysts

**Figure 4.**
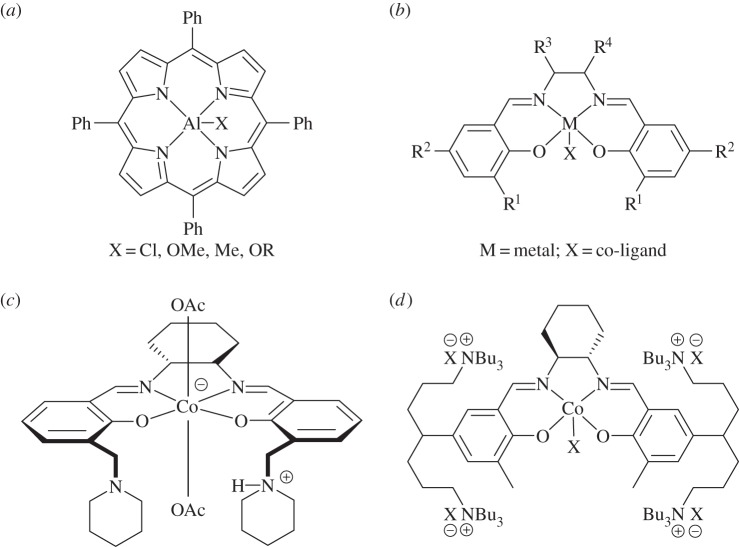
Bicomponent catalysts for CO_2_/epoxide copolymerization. (*a*) General porphyrin structure [32,53,63–65]. (*b*) General salen structure [55,57,66]. (*c*) Bifunctional salcy complex tethered to piperidinium moieties [67]. (*d*) Bifunctional salen complex tethered to quaternary ammonium groups [33].

These catalysts are used with an exogenous co-catalyst or, more recently, have the co-catalyst attached to the ancillary ligand scaffold [5,32,61–63]. The ligands are usually planar, tetradentate, dianionic compounds such as salens or porphyrins. The commonly used co-catalysts are ionic salts, such as PPNX and ammonium halides. The structures of typical catalysts in this class are illustrated in figure 4 [5,32,61–63].

The first well-defined homogeneous catalysts were metal(III) porphyrin complexes (e.g. figure 4*a*), which in combination with an ionic salt allowed the controlled ROCOP of CO_2_/epoxide [68]. There have subsequently been several studies to elucidate the influences of various ligand substituents and metal centres, which revealed that Co(III) centres coordinated by porphyrin ligands, substituted with electron-withdrawing groups, showed the best performances [32,53,63,64,69–75]. However, in general, metal porphyrin catalysts have lower activities and productivities compared with metal salen catalysts.

Catalysts based on salen ligands (figure 4*b*) are some of the most active catalysts for CO_2_/epoxide copolymerizations [5]. Darensbourg [5,22,62,76–79], Coates [15,31,55,80–87], Li [88], Nozaki [28,67,89–91], Lee [29,30,33,92–95], Rieger [66,69,96–98] and others have led the development of various [salenMX] complexes (where M=Cr(III), Co(III), Al(III)), many of which show very high rates and selectivities. Coates pioneered the application of chiral [Co(salen)] complexes as highly active CO_2_/PO ROCOP catalysts. This class of chiral cobalt salen catalysts has been extensively investigated by a number of groups and they now show outstanding levels of regio- and stereochemical control [2,31,55]. Indeed, they have been applied in the preparation of entirely new classes of stereocomplex polycarbonates, starting from racemic mixtures of epoxides [80–82,90].

The nature of the co-catalyst is also important; as mentioned PPNX salts are widely applied. Furthermore, the amount of co-catalyst is generally optimum at 1 equivalent (versus metal complex), as greater quantities reduce activity. This is proposed to be due to competitive binding (versus epoxide) at the metal centre [99,100]. Using more than 1 equivalent of co-catalyst also increases the rate of back-biting side reactions to form cyclic carbonate products [5,57,100,101]. The co-catalyst serves a number of roles in the catalytic cycle. It is proposed that the co-catalyst binds to the metal complex in order to complete an octahedral coordination geometry and, by trans-coordination, enhances the labilization of the initiating or propagating group [5]. Additionally, the co-catalyst may act as an external nucleophile which initiates polymerization. The precise mechanisms by which such catalysts operate are rather complex and not yet fully defined, but it is generally proposed that the rate-limiting step involves epoxide ring opening rather than carbon dioxide insertion [5].

**Figure 5.**
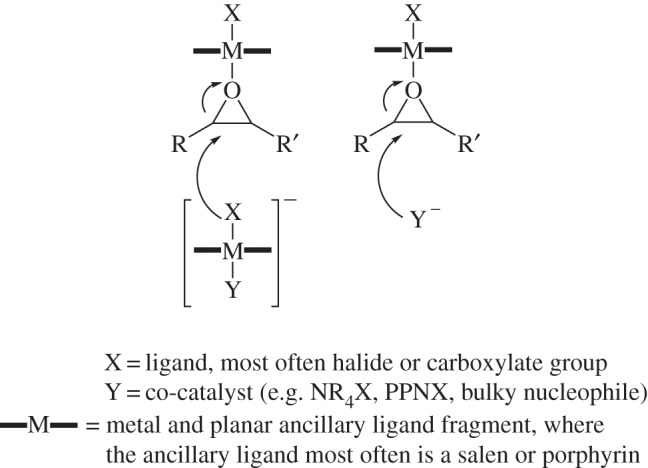
Possible pathways for epoxide ring opening by the bicomponent catalysts.

Recent kinetic studies have revealed that the polymerization rates, using metal salen catalysts, are typically dependent on catalyst concentration to a fractional order, usually between 1 and 2 [89]. This is indicative of pathways involving two metal complexes and/or dimerization in the rate-limiting step. Figure 5 illustrates the two different proposed pathways for epoxide ring opening. The fractional orders in catalyst concentration could be rationalized if both pathways are feasible and occurring concurrently. Significant work has also been carried out to investigate the coordination of epoxides to such catalysts [102–104]. The gas-phase binding of epoxides in Al(III) and Cr(III) salen and porphyrin complexes was studied by Chisholm and co-workers and found to follow metal Lewis acidity trends [103]. Darensbourg also showed that the ring opening of epoxides by a nucleophile was dependent on the metal–epoxide bond length and the binding enthalpies, not solely on the rate at which epoxide binding occurred [104]. It is worth noting that the coordination of co-catalysts changes the Lewis acidity of the metal centre and so would affect epoxide binding [105].

Recently, Nozaki and co-workers [89] demonstrated a theoretical model to predict the catalytic activity and selectivity in PO ROCOP using planar bicomponent catalysts. By comparing the difference between the dissociation energies for metal epoxide, carbonate and alkoxide intermediates, the preferred pathway at each stage of the catalytic cycle could be quickly estimated [89].

One challenge of exogenous co-catalysts is that, if the stoichiometry (versus catalyst) is not finely balanced, significant side reactions to produce cyclic carbonate can result. Furthermore, because the systems are bicomponent, low catalyst loadings are not generally feasible. Finally, the fractional orders in catalyst imply that dimerization may be necessary, thereby providing an entropic barrier to catalysis at low metal loading. Two strategies have been used to overcome these limitations: (i) the development of catalysts whereby the co-catalyst is attached to the ancillary ligand and (ii) the development of dinuclear salen catalysts.

Increased activities, tolerance and selectivities were observed by covalently bonding the co-catalyst moiety to the catalyst ligand, removing the need to add an exogenous co-catalyst source. Nozaki first reported this phenomenon for a Co(III) salen complex, substituted with piperidinium ‘arms’ (figure 4*c*) [67]. This catalyst showed very high selectivity for carbonate formation (more than 99%), a high turnover frequency (TOF) (250 h^−1^) and operated at room temperature, under 14 bar pressure of CO_2_ [67]. Lee subsequently also developed various bifunctional catalysts (figure 4*d*) substituted with ionic groups, which have shown some of the highest activities (TOF =26 000 h^−1^) ever reported and which are active under low catalyst loadings (1:25 000 catalyst : PO) [29,30,33]. Lu has also shown that a cobalt salen complex with a tethered quaternary ammonium salt is highly efficient (TOF up to 5160 h^−1^) for the terpolymerization of CHO with a range of aliphatic epoxides (such as PO) and CO_2_ [106].

### (b) Dinuclear or bimetallic catalysts

**Figure 6.**
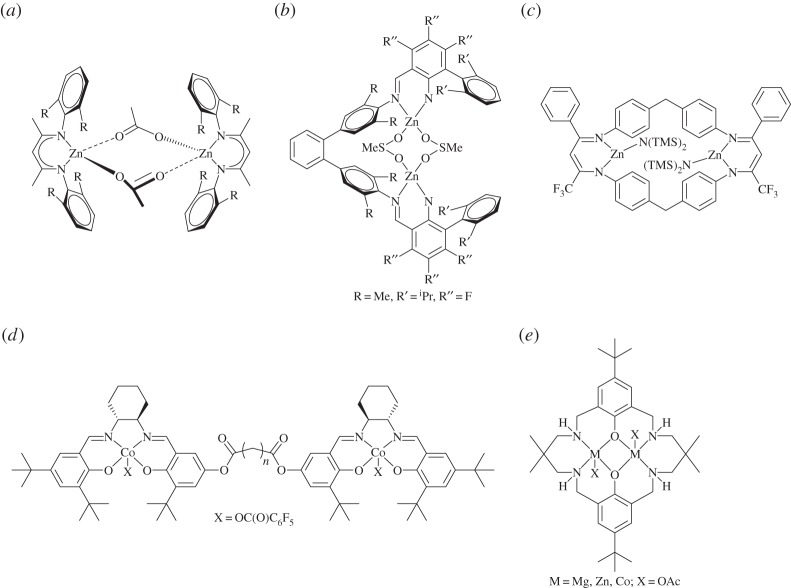
Dinuclear catalysts for CO_2_/epoxide copolymerization: (*a*) zinc BDI complex [56]; (*b*) zinc bis(anilido-aldimine) catalysts [108]; (*c*) tethered zinc BDI complex (TMS = trimethylsilyl) [109]; (*d*) tethered cobalt salen complex [91]; (*e*) dinuclear macrocyclic catalyst [110].

An important development for the field was the report from Coates, in 1998, of highly active zinc BDI catalysts [107]. These catalysts have shown good activity and selectivity for CO_2_/CHO ROCOP, at 50°C and 7 bar pressure of CO_2_. A wide range of [(BDI)ZnX] complexes have since been synthesized where ligand substituents exert a strong effect on activity (figure 6*a*) [107,111–114]. In a landmark paper investigating the mechanism, it was shown that the most active catalysts existed as ‘loosely associated’ dimers under the polymerization conditions [56]. Rate studies revealed that the order in zinc varied from 1.0 to 1.8, implicating dimeric active sites [56].

This study has inspired many others to develop dinuclear catalysts so as to increase activity and selectivity. Lee pioneered this with a series of bis(anilido-aldimine) Zn(II) complexes which showed high activities (TOF =2860 h^−1^) in copolymerization, at 1 : 50 000 [Zn] : [epoxide] loadings (figure 6*b*) [108]. The activity is sensitive to the *N*-aryl *ortho*-substituents and the fluorination of the aromatic rings significantly increased the TOF values [108]. Many researchers have investigated methods to ‘tether’ Zn(II) BDI catalysts [109,115–117]. The geometry, site and flexibility of the tether exert a significant influence over the activity, the best system for CO_2_/CHO being a dinuclear Zn(II) catalyst (figure 6*c*), which showed TOF values of 155 000 h^−1^, at 100°C and 30 bar [109,116,117].

Nozaki and Rieger have investigated the tethering together of two salen ligands (figure 6*d*) so as to target dinuclear catalysts [91,97]. When no co-catalyst was used, these catalysts operated via a bimetallic mechanism and showed improved performances (6–11 times better than the monometallic counterparts). Additionally, these catalysts were able to operate at low catalyst loadings (up to 20 000, epoxide/catalyst), supporting the notion that two metal sites may be involved in the pathway [91,97].

Our research group has focused on dinuclear catalysts coordinated by macrocyclic diphenolate ancillary ligands for CO_2_/CHO ROCOP [20,26,60,110,118–129]. The di-zinc catalyst (figure 6*e*) was the first catalyst to show promising activity under 1 bar pressure of CO_2_ [60]. Subsequently, we have explored a range of ligands, metals and co-ligands, including the first reports of active Fe(III) and Mg(II) catalysts for ROCOP [20,26,60,110,118–128]. Changing the metal centre significantly affects the polymerization rates, with the order of activity being Co(III) > Mg(II) > Fe(III) > Zn(II) using the same symmetrical ancillary ligand [20,26,60,110,118–128]. Kinetic studies of CO_2_/epoxide ROCOP, using the di-zinc acetate complex, showed a first-order dependence on CHO and catalyst concentrations and zero-order dependence on CO_2_ (1–40 bar) [124].

**Figure 7.**
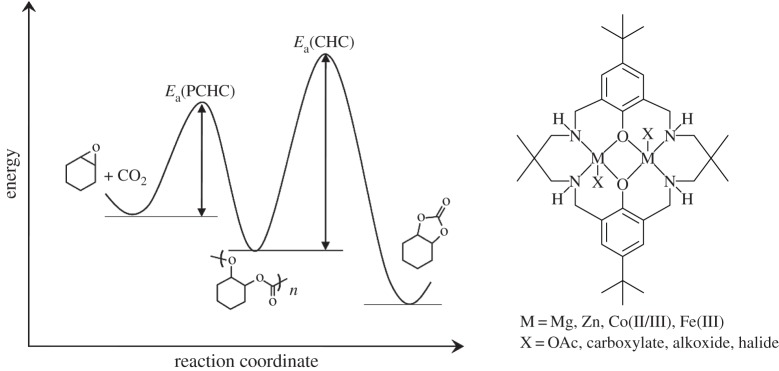
The different reaction energy barriers to the formation of PCHC versus cyclic carbonate. As part of the full kinetic analysis of the polymerization, the relative barriers were determined to be *E*_a_(PCHC) of 96.8 kJ mol^−1^ and *E*_a_(CHC) of 137.5 kJ mol^−1^. Adapted with permission from [124]. Copyright (2011) American Chemical Society.

Furthermore, the detailed analysis of the temperature dependence of the rate coefficients for both polymerization and cyclic carbonate formation enables a comparison of the relative barriers for polymerization (polycyclohexene carbonate, PCHC) versus cyclic carbonate formation (cyclohexene carbonate, CHC), the results of which are qualitatively illustrated in figure 7 [124]. It is apparent that the di-zinc catalyst shows a high selectivity for polymer formation due to the barrier to polymerization being approximately half the value for cyclic carbonate formation (*E*_a_(PCHC) of 96.8 kJ mol^−1^ and *E*_a_(CHC) of 137.5 kJ mol^−1^).

The rate law shows a zero-order dependence of carbon dioxide pressure (1–40 bar), which suggests that, under these conditions, the ring opening of the epoxide is rate-determining. The first-order catalyst dependence also supports the proposal of dinuclear propagation pathways. Although the catalyst is dinuclear, it also has two acetate co-ligands and therefore could potentially initiate two polymer chains per catalyst [123]. The polymerization kinetics show first-order dependences on both catalyst and epoxide concentrations, which implies that only one acetate co-ligand initiates polymerization, although more complex pathways cannot be ruled out on the basis of the rate law. This led to further investigations into the possible roles for the co-ligands.

**Figure 8.**
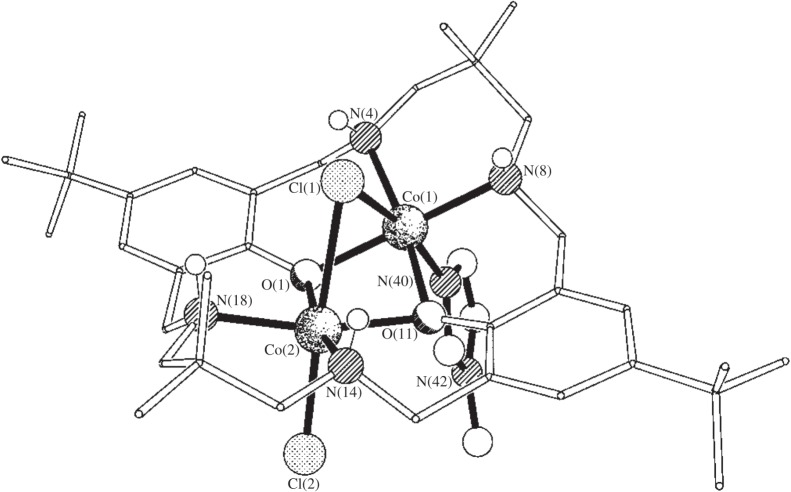
The structure, as determined using X-ray crystallography, of the di-cobalt catalyst [LCo_2_Cl_2_(methyl imidazole)]. The ‘bowl’ shape of the ligand is notable in the structure, as are the twofaces. The convex face refers to the outside of the ‘bowl’ (i.e. where Cl and MeIm are coordinated) and the concave face refers to the inside of the ‘bowl’ (i.e. where the bridging Cl ligand is coordinated). Adapted from [122].

**Figure 9.**
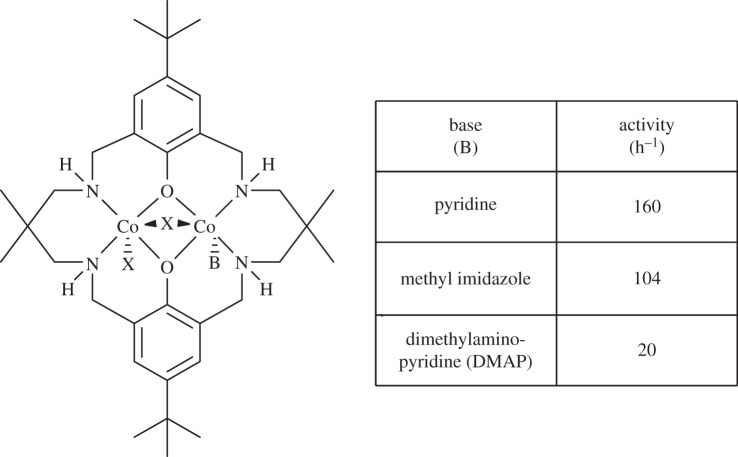
The structures of the di-Co(II) complexes and the corresponding activities in the copolymerization of CO_2_/CHO. Polymerization conditions: CHO : catalyst = 1000 : 1, 80°C, 1 bar CO_2_ [122].

A series of di-cobalt halide catalysts were prepared, with various neutral co-ligands being coordinated to them, including pyridine, methyl imidazole and DMAP (figures 8 and 9) [122]. The complexes all showed closely related solid-state structures, with the ancillary ligand adopting a ‘bowl’ conformation, whereby the NH substituents occupy the same ‘face’ of the molecule. In the concave face of the molecule, a halide ligand bridges the two cobalt centres. On the opposite convex face, a halide and Lewis base donor molecule coordinate to the cobalt centres (figure 8). It was discovered that the complexes showed rates strongly dependent on the nature of the donor ligand, with DMAP complexes having low activities and those coordinated by the weaker pyridine donor being equivalently active to catalysts without any co-ligand coordination. The finding that coordination of a single DMAP molecule could significantly slow the catalysis supported the notion that polymerization dominates from the convex face of the molecule and that two metals are involved.

A detailed spectroscopic and density functional theory (DFT) study also shed light on the polymerization pathways and key intermediates [123]. Using *in situ* attenuated total reflectance–infrared (ATR-IR) spectroscopy to characterize the reaction between di-zinc acetate complex and CHO showed that there were different environments for the acetate co-ligand. One of the acetate groups shows resonances consistent with attack at the CHO group, while the other has a resonance consistent with it maintaining a ‘bridging’ coordination mode between the two zinc centres.

The DFT study, carried out in solution, using appropriate functionals to model the polar environment, and using the complete catalyst structure, also substantiated the proposal that, although both metals are involved in propagation, only one of the two co-ligands initiates and propagates polymerization. The remaining co-ligand remains coordinated to the metal centres and mediates the polymerization process. Indeed, it was revealed by DFT that the polymer chain ‘switches’ coordination site between the two metal centres with each monomer insertion (epoxide or carbon dioxide). The chain shuttling is counter-balanced by an equal but opposite change in coordination site for the acetate co-ligand. It was shown that, by changing the co-ligand, it was possible to alter the rate of polymerization and DFT could be used to predict activity [123].

**Figure 10.**
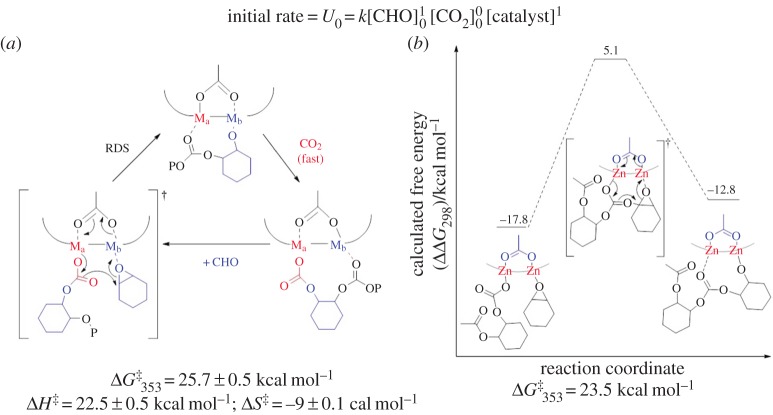
Comparison of the experimentally determined free energy barrier (*a*) to polymerizationwith the value determined by DFT (*b*) [123]. (Online version in colour.)

The chain shuttling mechanism implies that there are distinct roles for the two metal centres, as sketched in figure 10. Thus, one of the metal centres (illustrated in blue) coordinates the epoxides, while the other centre (illustrated in red) inserts carbon dioxide. The requirements for these processes are distinct, with epoxide coordination being accelerated by Lewis acidic/electrophilic metal centres, while the carbonate formation and attack step is favoured by metals showing labile carbonate groups. Such a mechanism would be expected to be improved by having distinct metals serving the two roles and thus provided an impetus to study heterodinuclear catalysts.

**Figure 11.**
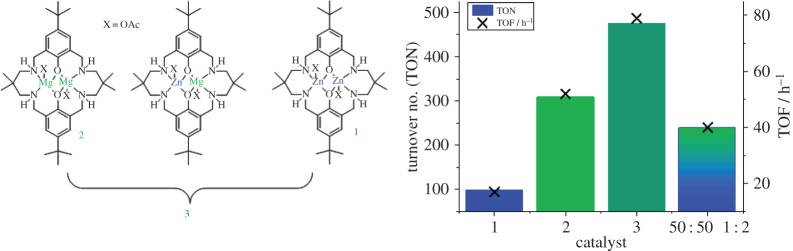
The structures of di-zinc (1), di-magnesium (2) and the heterodinuclear mixed catalystsystem (3) which were compared for the ROCOP of CO_2_/CHO [120]. (Online version in colour.)

In 2014, our group reported the first example of such a system [120]. The catalyst is a mixture of compounds, including both homodinuclear complexes and the heterodinuclear complex. Figure 11 compares the activity and productivity of this catalyst mixture, the homodinuclear complexes alone and in a 50 : 50 combination. It is clear that the mixture containing the heterodinculear catalyst shows an activity that is greater than either of the homodinuclear complexes or combinations of them. This finding provides the first evidence, albeit of a mixture of components, that heterodinuclear complexes are worth investigation in this field of catalysis [120].

## 5. Conclusion

The ROCOP of carbon dioxide and epoxides provides a useful means to reduce pollution, to consume carbon dioxide and to produce aliphatic polycarbonates. Low-*M*_n_, hydroxyl-end-capped, polycarbonate polyols are emerging as useful materials for the production of higher polymers, notably polyurethanes. There remain significant opportunities and challenges to understand and optimize the material properties of the polymers depending on the catalyst and raw materials available.

Catalysis plays a key role in the success and scope of this reaction. The selection of the catalyst is central to being able to control features such as the rate, productivity, selectivity, regio-/stereo-chemistry, polymer molecular weight, polymer end-groups and polymer composition and to produce block copolymers. So far, several successful heterogeneous and homogeneous catalyst types have been studied. A common theme is that the pathways are often proposed to occur via dinuclear or bimetallic routes, whereby one metal activates the epoxide, while the other metal provides the nucleophile (carbonate) to attack and ring-open the epoxide. Thus, recently, a number of improvements to catalyst rate and selectivity have been achieved by targeting structures that optimize the coordination chemistry of dinuclear catalysts. The discovery and further development of dinuclear and bimetallic homo- and heterogeneous catalysts is an important area for future research.
